# Tracking the mutual shaping of the technical and social dimensions of solar-powered mosquito trapping systems (SMoTS) for malaria control on Rusinga Island, western Kenya

**DOI:** 10.1186/s13071-014-0523-5

**Published:** 2014-11-18

**Authors:** Prisca A Oria, Alexandra Hiscox, Jane Alaii, Margaret Ayugi, Wolfgang Richard Mukabana, Willem Takken, Cees Leeuwis

**Affiliations:** Knowledge Technology and Innovation Group, Wageningen University and Research Centre, Wageningen, The Netherlands; International Centre for Insect Physiology and Ecology, Nairobi, Kenya; Laboratory of Entomology, Wageningen University and Research Centre, Wageningen, The Netherlands; Context Factor Solutions, Nairobi, Kenya; School of Biological Sciences, University of Nairobi, Nairobi, Kenya

**Keywords:** Malaria, Co-evolution, Socio-technical, System innovation, Solar, Mosquito trap, Feedback, Community, Kenya

## Abstract

**Background:**

There has been increasing effort in recent years to incorporate user needs in technology design and re-design. This project employed a bottom-up approach that engaged end users from the outset. Bottom-up approaches have the potential to bolster novel interventions and move them towards adaptive and evidence-based strategies. The present study concerns an innovative use of solar-powered mosquito trapping systems (SMoTS) to control malaria in western Kenya. Our paper highlights the co-dependence of research associated with the development of the SMoTS technology on one hand and research for enhancing the sustainable uptake of that very same intervention within the community on the other.

**Methods:**

During the pre-intervention year, we examined the design, re-design and piloting of a novel technology to generate lessons for malaria elimination on Rusinga Island. Initial ideas about many technological necessities were evaluated and re-designed following feedback from various sources, including technical and social research as well as broader interactions with the social environment. We documented the interlocking of the multiple processes and activities that took place through process observation and document reviews. We analysed the data within the conceptual framework of system innovation by identifying mutual shaping between technical and social factors.

**Results:**

Our findings illustrate how various project stakeholders including project staff, collaborators, donor, and community members simultaneously pursued interdependent technological transformations and social interests. In the ongoing process, we observed how partial outcomes in the technological domain influenced social events at a later phase and vice versa.

**Conclusions:**

Looking at malaria intervention projects employing novel technologies as niches that may evolve towards system innovation, helps to reveal interrelations between the various technical and social aspects. Revealing these interrelations requires a different role for research and different perspective on innovation where innovation is more than the technical aspects. This approach therefore requires that research is designed in a way that enables obtaining feedback from both aspects.

## Background

Technology is affected at a fundamental level by the social context in which it develops [1,2]. Adopters of technology may be signing up for far more – politically, economically, even culturally, as well as technically – than appears at first sight [1,3].

While social scientists working in public health have devoted much attention to the effects of technology on society, they tended to ignore the more fundamental question of what shapes the technology in the first place [1,4,5]. Some progress to change this has been made over the years [6,7]. In relation to malaria control, while researchers rarely investigate the social processes that shape malaria control innovations before implementation, there is increasing focus on making improvements to progressive forms of the interventions based on implementation research insights on human responses to predecessor innovations [8,9] or on the bio-physical conditions that determine their effectiveness [10,11]. In the past, it has been shown that challenges of community engagement can undermine research, even in studies where ethical issues have been addressed, as was the case with the abandoned trials in Cameroon and Cambodia of tenofovir as pre-exposure prophylaxis against HIV infection [12]. Learning from these experiences, public health technology developers strive to anticipate public acceptance actively by including social contexts in the design and development of their innovations. An innovation, then, is the effective combination of new technology (hardware) and the novel forms of social organisation (orgware). This emphasises the interdependence of the social and technical aspects of an innovation because the hardware does not fulfil societal functions on its own but in association with human agency, social structures and organisations [3,13,14]. Despite advances in the technology assessment field in general, the social issues associated with new technologies are still not fully considered [15,16]. For understanding the efficacy of an innovation in context it is necessary to understand the interaction between the technical and social phenomena.

The key research and development goal in malaria control is to define an agenda to sustain and improve the effectiveness of currently available tools and to develop new vector control tools that can be used to interrupt transmission in environments or at intensities that existing tools cannot reach [17]. Studies have shown that during the design phase, technology actors usually focus on developing, testing and optimising technology but often neglect embedding the technology in broader societal goals, or leave it to a later pilot stage [2,18]. However, embracing bottom-up approaches that engage end users from the outset in research and development have the potential to bolster vector control and move it towards adaptive and evidence-based strategies that vary in space and time depending on local conditions [19]. Sustainable innovations development therefore requires interrelated social and technical change [2]. This is necessitated by the fact that social impacts are not side effects but core dimensions of new technology and technological development, they are a function of the co-production of technology and society [15]. In this way, an innovation project is best advanced by engaging the end users and working in partnerships to generate shared knowledge and solutions relevant to the local context, in addition to optimising the physical functioning of the hardware. Interventions become embedded through the manipulation of these contextual factors that enhance the uptake, performance and sustainability of the intervention [18,20].

**Figure 1:**
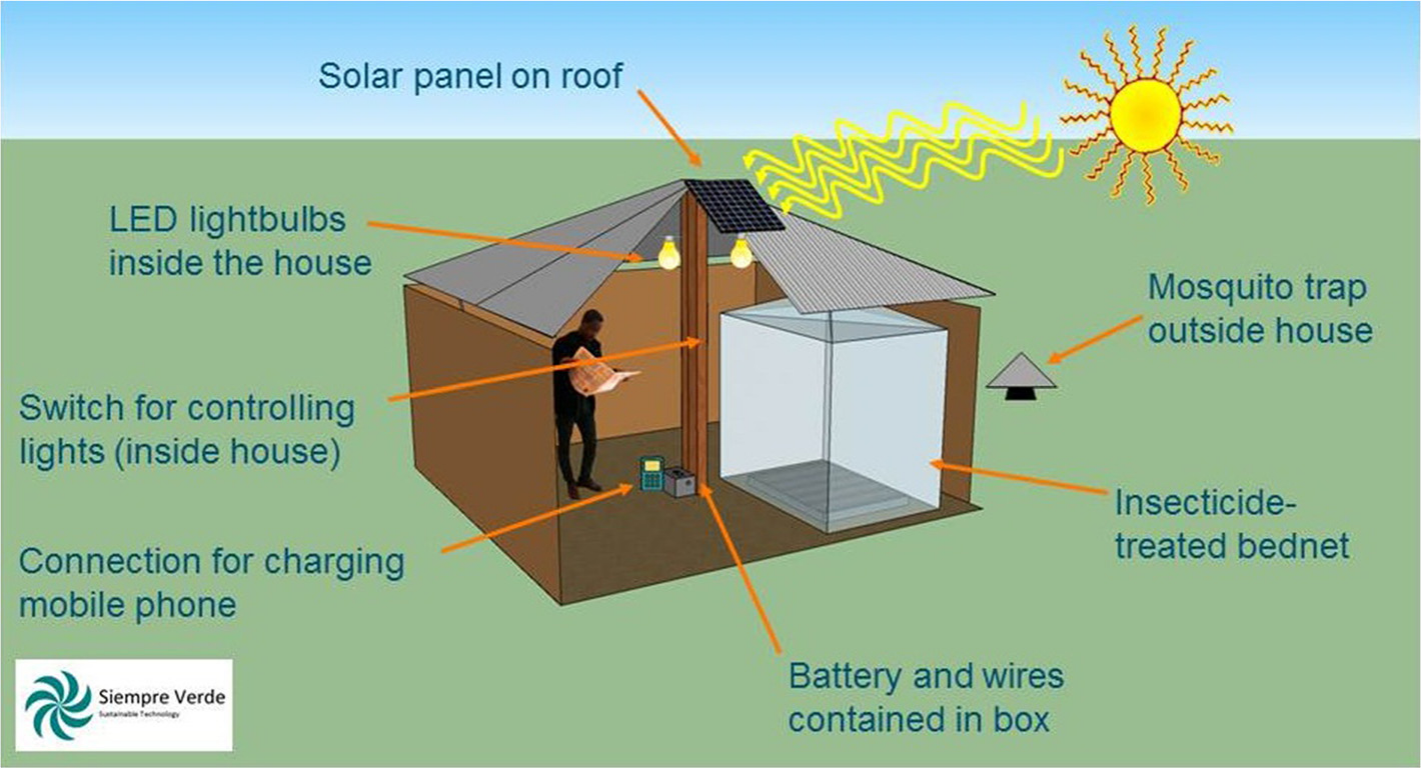
**Model house with SMoTS installed.**

In 2012, we launched a community-based malaria control intervention project using Solar-Powered Mosquito Trapping Systems (SMoTS) on Rusinga Island, western Kenya – the SolarMal Project. The use of novel technology underpinned all areas of the project; from the optimisation of chemical baits to attract mosquitoes, to the design of a new mosquito trap and the installation of solar panel systems to provide power to run the traps [21]. A SMoTS is being distributed to each homestead on Rusinga Island. A homestead is a single fenced-in house or group of houses occupied by one nuclear or extended family respectively. An installed SMoTS consists of a solar panel mounted on the roof of a house, a battery, a battery box with a USB mobile phone charging port, two Light-Emitting Diode (LED) light bulbs and a mosquito trap hung outside the house (Figure 1). The original concept of a SMoTS included house lighting as an additional benefit but the inclusion of a USB mobile telephone charging capacity was incorporated at a later stage of development. Each homestead receives one SMoTS. In a homestead with more than one house, members agree through consensus on which house to install the SMoTS.

The project roll-out uses a variation on the stepped wedge trial design, termed the hierarchical design. The intervention implementation began at one randomly selected homestead and expanded randomly until a cluster (defined in this study as a composition of 50–60 homesteads) with the intervention was created. Neighbouring clusters then received the intervention until a metacluster (defined in this study as a composition of nine clusters) was intervened. The intervention implementation then progressed into clusters and metaclusters in a second geographically distinct location, then a third, fourth, fifth, etc., and this will continue until the whole island is covered [21].

The main objective in developing the study design was to ensure that the roll-out of the intervention proceeded in such a way that the project was able to maximise the possibility of detecting an effect of the intervention on malaria clinical incidence and parasite prevalence. A step-wise approach was needed due to logistics of installing the systems and to enable measurement of the time taken for the intervention to be effective in any area. Randomisation at the homestead level could create contamination of effectiveness measures by mosquitoes entering the intervention area or by extending the effect of the intervention to neighbouring houses, thus protecting houses beyond the homestead in which the SMoTS was installed and effectively reaching a situation where the entire study area is intervened with no remaining control area for comparison. The roll-out commenced in June 2013 and it will take an estimated two years to reach complete coverage. Boundaries of intervention areas are not the same as village boundaries due to variation in village sizes and the need to create intervention areas of the same number of homesteads. This therefore means that parts of villages on the island receive SMoTS ahead of others.

Our analysis focused on the design, re-design and piloting of the innovative approach to controlling malaria largely before its implementation had started. We systematically documented and analysed how the mosquito trapping technology and related social contexts mutually shaped each other and how this mutual shaping impacted design and re-design of the intervention. This paper highlights the co-dependence of the research associated with the development of the SMoTS technology on one hand and the research for enhancing the sustainable uptake of that very same intervention within the community on the other. In our analysis we demonstrate how system innovation theory helps to provide insights into how a promising malaria control intervention evolves and matures through an interaction between technical and social phenomena.

### System innovation and the co-evolution of technology and society

System innovation theory suggests that system innovation happens through experimentation in socio-technical niches which compete with other niches and the existing regimes. New technologies require the adaptation of socio-technical regimes [22,23]. The experimentation that occurs is a mechanism to adapt to a broader system and must take place in a protected environment that enhances the chances of the new technology prospering even when faced with competition from other technology and associated actors and social interests.

In working towards system innovation, an innovative idea such as this project needs not only to involve technological substitutions, but also changes in social elements [13,24,25]. The end result is that mature incumbent technologies and the existing technological regime are well attuned to each other as a result of a long process of incremental co-evolution [26].

When talking about societal change it is important to acknowledge that human agency, strategic behaviour, and social struggles are important but situated in the context of wider structures [13]. Actors interact within the constraints and opportunities of existing structures, while simultaneously acting upon and restructuring these systems. Structures not only constrain but also enable action, making action possible by providing coordination and stability. However, socio-technical reconfigurations do not occur easily because the elements in the configuration are aligned to each other. Radically new technologies have a hard time in breaking through because the various networks are aligned to the existing technology [27].

Co-evolution takes place when two or more variables of the system affect and essentially create each other, although their different variables may operate at different scales. Social systems thus adapt themselves to changing technical systems, as well as the converse [13]. Social shaping of technology is accompanied by technical shaping of society.

Using the above perspective, we regard this project as a niche level activity, aimed to enhance the success of the intervention in both the health and energy regimes. The challenge for the project is to create social and technical novelties and learn how they can be made to work in practice by involving real life stakeholders in their specific context. Thus, in order to effectively combat malaria, the new SMoTS technology needs to become effectively adapted and linked to both a dynamic social and the relevant bio-physical environment, whereby it is relevant to acknowledge that these environments themselves may be influenced deliberately as part of the innovation process. In other words, the SMoTS will eventually have to ‘work’ socially, for instance, in the sense that they are accepted and supported by behaviours of individuals and organisations, and they will have to ‘work’ technically, in the sense that they actually capture sufficient mosquitoes in the prevailing geographical and bio-physical conditions of Rusinga Island.

## Methods

This social research was carried out within a multidisciplinary team. Research into the interaction between technical and social phenomena in the development of malaria control innovations requires a strategy that is both rich in context and can track developments over time [13]. The focus is on documenting the interlocking of multiple processes and activities. This article explores the pre-intervention year of the development of a community-based innovative malaria control project which employed the methodology of action research. Beginning April 2012 until April 2013, we examined the design, re-design and piloting of a novel technology to control malaria.

The process of piloting SMoTS in the field took place over a six week period in 2012. The aim of the pilot study was to ensure that the SMoTS functioned from a technical perspective and to assess residents’ perceptions of the SMoTS before placing a large order for components. As part of the piloting, the project installed complete SMoTS in the study community to test and evaluate their performance and community perceptions. A total of 18 SMoTS were installed in randomly selected homesteads. Before the piloting, representatives of the selected homesteads were invited to an orientation session, during which they were informed of the reasons for and duration of the pilot, how SMoTS work, and how to care for SMoTS, among others.

During the piloting, the project installed nine 20-Watt and nine 30-Watt solar panels in the selected homesteads. The project piloted four different types of bulbs: in each household we installed two different types of LED bulbs, one brighter than the other. All bulbs were three Watts and white but their brightness and physical size differed. The piloting was to determine whether a 20-Watt panel would provide sufficient energy to run the SMoTS, or if a 30-Watt system was required. The performance and compatibility of the battery and bulbs within the households was also assessed. Technical assessments included checking the voltage of the batteries after a night of use and checking that the lights and trap functioned during all nights. Project staff also held informal conversations in houses that received a pilot SMoTS in order to capture occupants’ perceptions of the installed SMoTS. Essentially, the project wanted to ensure cost-effectiveness without compromising the research i.e. to ensure sufficient power supply for operating the mosquito trap yet cognizant of the practical immediate interests to households, such as lighting and phone charging. The findings formed the basis for the larger procurement order.

During this piloting period, the project community engagement mechanisms were also being refined and implemented. The research employed document reviews and ethnographic methods of process observation.

### Study site and population

The trial targets all residents of Rusinga Island, an island in Lake Victoria, western Kenya. The island is extensively deforested and generally rocky with limited vegetation cover [28]. Rusinga has a diverse topography, ranging from flat areas near the shoreline to a central hill. Although malaria is transmitted throughout the year, intensity can vary greatly according to seasons. The area experiences long rains between March and June and short rains between October and November, although the interval of the rains has become unstable in recent years [28].

As per a census implemented at the end of 2006 during the establishment of a demographic surveillance system, Rusinga Island had 24, 000 inhabitants [29]. Residents are primarily engaged in fishing in Lake Victoria, small-scale trading and subsistence agriculture [28]. The local language is Dholuo. Most houses on the island have walls made from mud or corrugated iron, with corrugated iron roofs. Lake Victoria is the main source of water for the islanders. The lake is used for fishing, washing clothing and dishes, and bathing. Latrine usage is low. Except for a few businesses, guest houses, and NGO offices, running water and mains electricity are largely untapped. Generators are occasionally used to pump water, operate flour mills, run mobile phone charging businesses or power speaker systems for events such as church services and religious meetings. Prior to 2012, most Rusinga inhabitants used kerosene lamps as light source and had their mobile phones charged at commercial centres.

### Data collection and analysis

The concept for the intervention arose following the discovery of synthetic odours that attract malaria mosquitoes by mimicking human odour [30]. After the discovery, researchers started thinking about how to implement the technology in an actual field setting. This led to the development of ideas about whether it would be possible to use traps baited with the synthetic odour and carbon dioxide to lure and capture mosquitoes. Electricity would be required to power fans which could suck mosquitoes into the traps and the power could be generated through solar energy. During the pre-intervention year initial ideas about many of these necessities had been developed but it was during this time that they were evaluated and re-designed following feedback gained from various sources, including technical and social research as well as broader interactions with the social environment. The main aim of the re-design was to customize the intervention to the local setting of the trial community.

**Figure 2:**
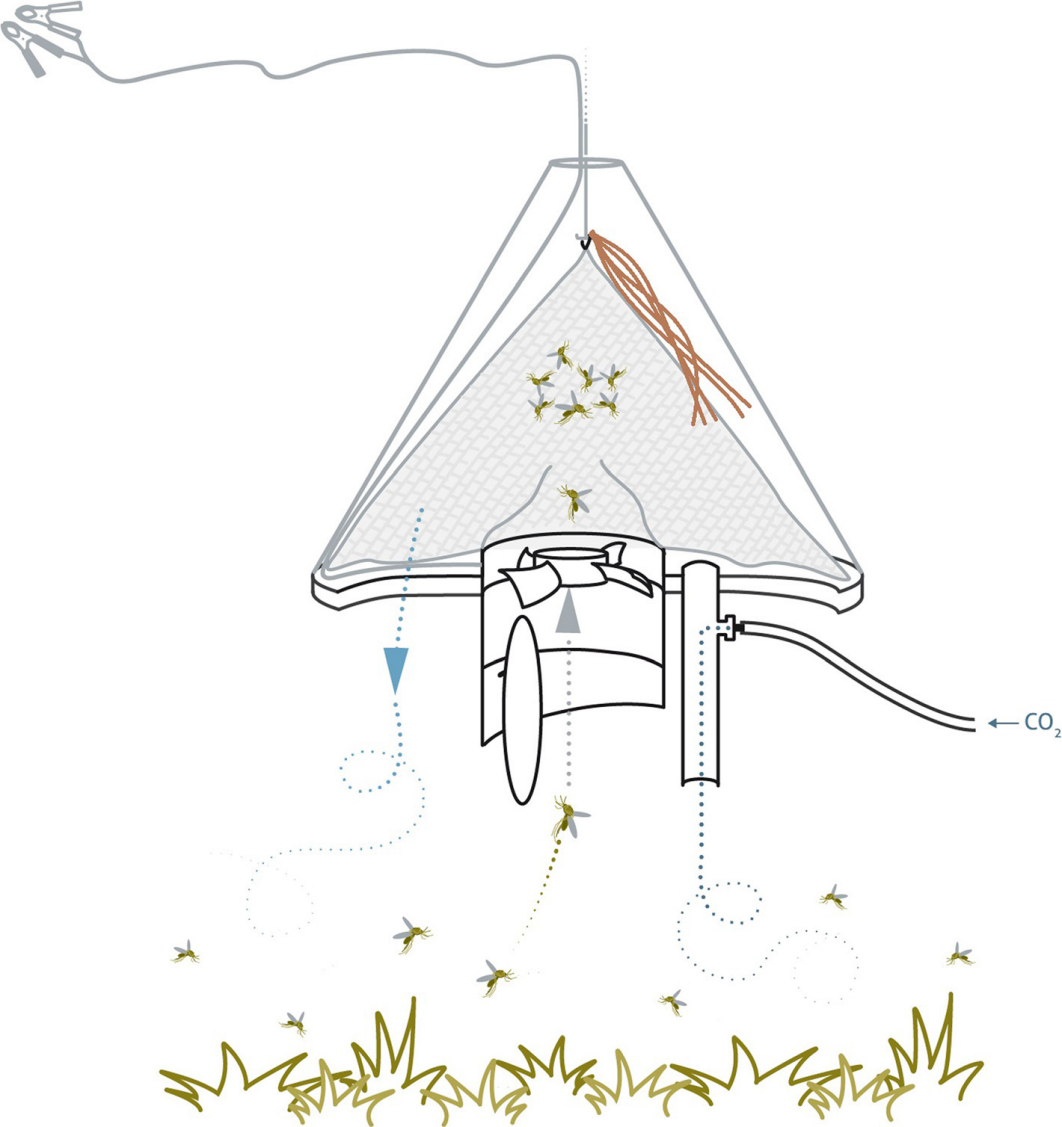
**Cross-sectional diagram of the** ***Suna*** **mosquito trap (source: Hiscox** ***et al. Malaria Journal*** **2014 13:257 doi:** **10.1186/1475-2875-13-257).**

The mosquito traps (*Suna* traps) operate according to a counter flow mechanism and are designed to collect mosquitoes outdoors prior to house entry [21]. Chemical odours placed on nylon strips attract mosquitoes to the trap. A mosquito nearing the trap is sucked through a ventilator into a bag inside the trap. Trapped mosquitoes cannot escape and they eventually die due to lack of water and food. The *Suna* trap has been described elsewhere [31]. Figure 2 shows a cross-sectional diagram of a *Suna* trap.

Data presented here were collected in an action research mode. Over a period of one year we convened several project meetings with community members, three meetings with members of the project community advisory board (CAB), and several meetings with members of a community-based organisation (CBO). The aim was to understand the research subject wholly within its social context.

We collected field notes during meetings and expanded these on an MS Word 2007 (Microsoft, Washington, USA) file afterwards. Observational notes and reflexive dialogues were also hand written and expanded on an MS Word 2007 file. The progression from data collection to interpretation was intended to be reflexive. We analysed the data within the conceptual framework of system innovation by identifying mutual shaping between technical and social factors. We noted changes in technical and social designs of the intervention, put them on a timeline and reconstructed the rationale for the changes, and related them to technical or social considerations. We also monitored the effect of the changes on the technical and social refinements on the design and re-design of the intervention.

### Ethical considerations

The SolarMal study was approved by the Kenya Medical Research Institute Ethical Review Committee (KEMRI-ERC NON-SSC No. 350). After the study was explained to the households in the local language, written informed consent was obtained from them prior to enrolment.

## Results

### Changes to technology design

Changes to the technology design included removal of carbon dioxide from the blend, trap improvements and re-design of the electricity provision system. Table 1 summarises these changes, provides a timeline of when they occurred, feedback that necessitated them and their consequences.

**Table 1:** **Mutual shaping of technology and social contexts of the intervention**

**Period**	**Event/decisions**	**Feedback**	**Consequences of feedback**
Jan-March 2012	Commenced trap development with the introduction of Mosquitito trap™.		
April-June 2012	Continued trap development with the first *Suna* trap.	Solid metal cone introduced because fabric absorbed the odours and consequently reduced trap efficacy.	More durable.
		Fabric base replaced with flexible plastic mesh base.	
July-September 2012	Continued *Suna* trap development with the replacement of metal cones with plastic ones and plastic mesh base with a rigid one.	Metal cones are potentially attractive to thieves who could sell them to scrap metal dealers.	Lower unit cost for SMoTS.
		Plastic cones are cheaper than metal cones.	Rigid plastic base to increase durability but found to reduce airflow and performance.
July-September 2012	Complete SMoTS installed in 18 households for piloting. In nine households 20-Watt systems were provided and in the other nine, 30-Watt systems were provided. Also, four different types of bulb were provided.	Performance of various components and community perceptions of SMoTS.	Decision on final SMoTS components: 20-Watt systems and brighter bulbs selected for the intervention.
		Estimates of lengths of electrical cable needed per house.	
July-September 2012	Removal of carbon dioxide from the blend.	Logistical challenges with procuring and distributing molasses to households.	Discontinued mobilisation of women’s groups that were being mobilised to distribute molasses for fermentation.
		Time constraints with regard to project timelines.	
		Need for more intensive training to households on replacing molasses on a daily basis and concerns about adherence.	
		Cost of procuring molasses.	
October-December 2012	Finalised trap development with the modification of plastic base with fine grid of holes to increase airflow (Figure 3).	Rigid plastic base with fine grid of holes.	Increased airflow and performance with greater durability than a fabric base.

#### Re-design of the mosquito trap

The components of the SMoTS were designed and developed through a collaboration of a network of actors and institutions. Trap development began with the Mosquitito™ Trap which was already produced and sold by Biogents AG (Regensburg, Germany). The Mosquitito™ Trap is used to capture *Aedes* mosquitoes that are potential vectors of diseases such as Chikungunya and dengue viruses, among others. The Mosquitito™ Trap was modified to create the final *Suna* trap which is now used for the intervention.

From April to June 2012, the first prototype *Suna* trap was developed. *Suna* is the Dholuo word for mosquito. In this prototype the fabric base and fabric cone of the Mosquitito™ trap were replaced with flexible plastic mesh base and metal cone because experiments with the fabric Mosquitito™ trap showed that *Anopheles gambiae* catch sizes decreased by around 20% over time under semi-field conditions and there were concerns about the durability of a fabric trap. The fabric was suspected to absorb odours from the bait, thus leading mosquitoes to approach the trap not only from the lower side where they would be sucked inside, but also from the upper side where there was no trap entry point.

**Figure 3:**
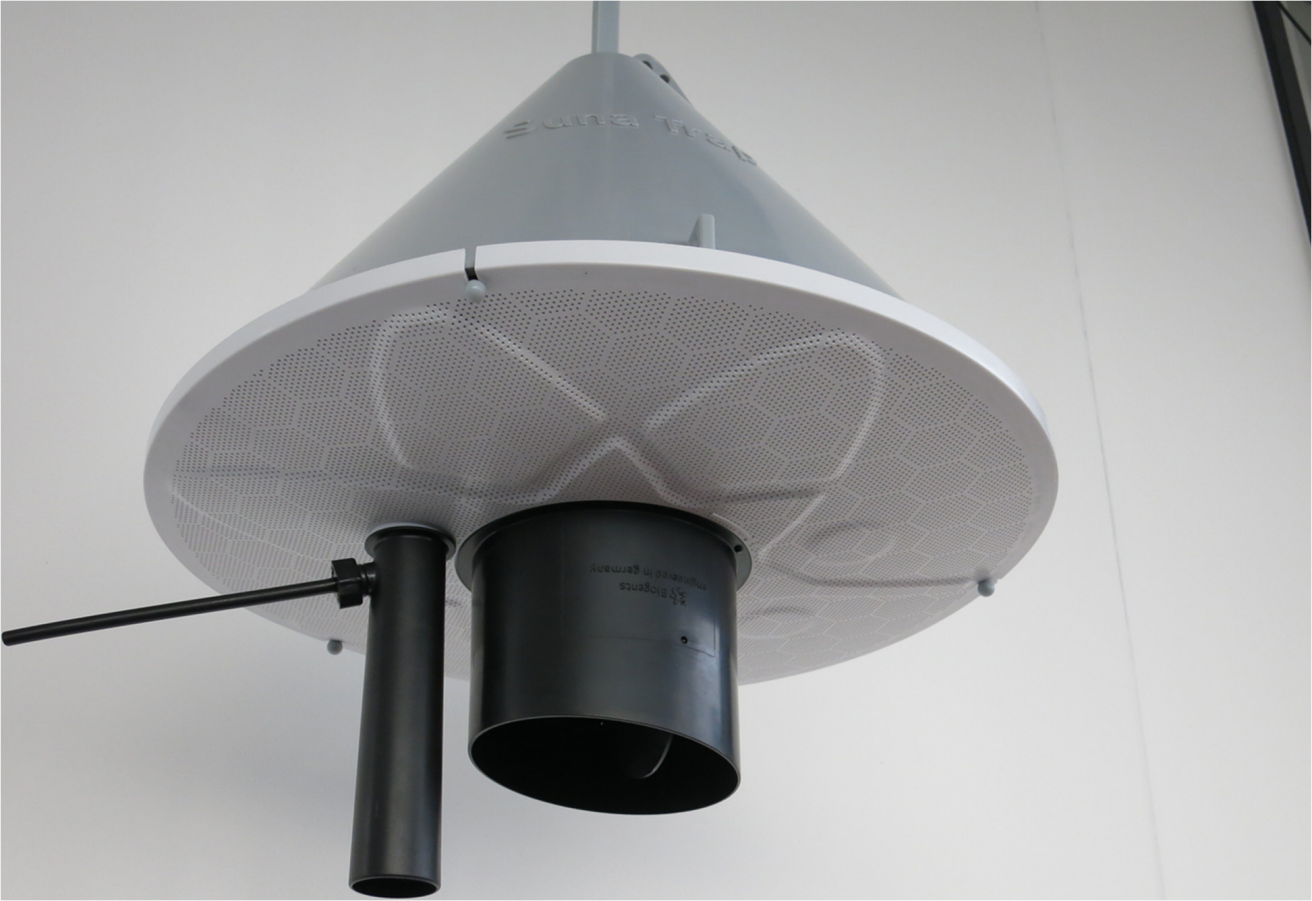
***Suna*** **trap with a plastic base with fine holes.**

Between July and September 2012, the second *Suna* trap prototype was developed using a more durable rigid plastic base with large air holes and a metal cone. Under semi-field conditions, comparisons of this trap against the first prototype associated the solid plastic base with a 60% reduction in *An. gambiae* catch size. From October-December 2012, the *Suna* trap was modified with a plastic base with a fine grid of holes (Figure 3) to increase air flow to a rate similar to that of the flexible plastic mesh base. The cone was also re-designed so that it could be made from plastic rather than metal to reduce cost and risk of theft. Community members were concerned that metals are more attractive to thieves because there is a ready market for scrap metal. The *Suna* trap in its final form is now sold by Biogents AG (http://www.biogents.com/cms/website.php?id=/en/traps/biogents-trap-systems/bg_suna.htm).

#### Re-design of the electricity provision system

Findings of the piloting with regard to the solar panel, battery and bulb performances suggested that a 20-Watt solar panel provided sufficient energy to simultaneously run a *Suna* trap, charge a mobile phone and light the two LED bulbs. Thus, the project procured 20-Watt solar panels for the intervention. In addition, we noticed dead insects inside some of the bulbs. Ultimately one brand of bulbs was preferred because it gave the brightest light and insects could not get inside.

Cost limitations also shaped many decisions taken in the development of the SMoTS. The cost of components, particularly the solar panels and battery, were important determinants of the end functions of the system.

A report compiled from routine informal conversations with household members during the period they had a SMoTS for piloting revealed that households expressed relief with regard to reduced expenses on kerosene for lighting houses; they either did not need kerosene at all or only needed to buy small quantities for lighting houses that did not have SMoTS. Even though the pilot group could use the systems to charge their mobile phones if they bought a USB cable, only four households bought a USB cable and used the system to charge their phones. During this period, in contrast with the present situation, USB cables were not sold in the vicinity of Rusinga Island. People who charged their phones were excited about having battery time on their phones all the time and saving money they would have otherwise spent on charging their phones at commercial centres.

#### Removal of carbon dioxide from the odour blend

Carbon dioxide plays an important role in the host-seeking behaviour of blood-feeding mosquitoes. The project initially planned to use a mixture of organic volatiles (ammonia, lactic acid, tetradecanoic acid, 3-methyl-1-butanol, butan-1-amine), impregnated on to strips of nylon, supplied in combination with yeast and molasses-generated carbon dioxide [32]. The yeast and molasses mixture would need to be replenished every day in order to provide carbon dioxide to the trap during every night of trapping. The project therefore needed to develop a molasses procurement and distribution system to ensure all 4000 plus homesteads on the island had a supply of molasses every day. The project started engaging women’s groups which were based all over the island to brainstorm on a mechanism for distributing molasses to all homesteads. The project would need to build a central store for molasses on the island from where women from different groups would on a weekly basis collect and distribute it to homesteads for daily replenishment.

Due to increased awareness of financial and logistical challenges related to continuously procuring and distributing molasses in quantities large enough to supply all homesteads on the island on a daily basis, and the unsustainable aspects of molasses provision, the decision was taken to remove carbon dioxide from the blend and replace it with a synthetic mimic [33]. Because the 5-component odour bait and carbon dioxide mimic was expected to attract and remove a constant fraction of the malaria vector each day, it was considered that the continuous presence of the odour-baited traps was more important in controlling malaria than maximum daily efficacy. The new odour combination was released from small nylon strips suspended inside the cone of the *Suna* trap [31].

Delivery of the odour-bait from nylon strips is the most effective way of producing odour baits at present [34]. In addition, nylon is locally available and is relatively cheap. At present the odour bait is replaced every three months, but it is expected that research and development work will lead to the creation of odour baits which last longer [35].

### Changes to the social organisational design of the intervention

In order to gain and maintain the support of communities and organisations on the island the project had to carefully operate and adapt its implementation strategies on several occasions. While in the early stages the islanders easily showed enthusiasm for the project, the electrification aspects in particular, later on a number of sensitivities occurred. These related, for example, to issues about who should represent the community in the project organising team and about whom should receive SMoTS and in which order the systems should be rolled out.

#### Community engagement: from a community-based organisation (CBO) to community advisory board (CAB)

During the initial stages the project worked with members of an already existing community-based organisation (CBO) as a link between the project and the community. However, the project’s engagement with the CBO was characterized by challenges and tensions related to differences in priorities between the CBO and project, the extent to which community members perceived the CBO to represent and reflect community aspirations, and competition between the CBO and other community groups. This hampered initial efforts to foster effective relationships between researchers and the research community.

Based on feedback from meetings with community leaders and members, the project realised that while the CBO’s liaison role may fit other on-going community-based research in Rusinga, this synergy did not necessarily cut across projects. This led to conceptualisation of a community advisory board (CAB). This group would provide advice and act as a resource for the project team on issues of community engagement. Considerations for membership into the board recognised the expertise of the members’ knowledge of the community of Rusinga. The board would interpret the community responses to the project staff and interpret the project to the community. The project team worked with project stakeholders including healthcare workers, church representatives, government administrators, representatives of the fishing community, women and youth representatives, non-governmental and community based organisations to identify key sectors of the community to be represented. The people included in the list were either nominated or elected by community members to represent a section of the community. The above mentioned CBO was invited to join the CAB, in recognition of their role representing a specific group of the island community. The process led to the development of a list of 16 persons who constituted the project CAB. Membership of the CAB is broad-based with representatives drawn from government administration, Ministry of Health, churches, beach workers, women, the youth, the education sector, non-governmental organisations, community-based organisations, political sector, and lay community members. The overriding objective was to have a group that is representative of all sectors of the community so that whenever the project obtains the viewpoints of the board, ideas which are representative of the residents of the island are heard. During their first meeting the CAB members elected an executive committee comprising of a chairperson, vice-chairperson, secretary, and treasurer.

The project then organised a workshop to orient and train the board members to provide them with a broadened understanding of the project. During this event CAB members were trained regarding their functions and protocol-related awareness. Active CAB participation in the intervention process was encouraged.

The CAB immediately became critical when the project engaged it in discussions on how to select a house to install a SMoTS in homesteads with more than one house (see Selection of house to install the SMoTS). Members of the CAB were also instrumental in devising a strategy to pick a sequence to follow in rolling-out SMoTS to different clusters and metaclusters. They provided feedback during simulation of a roll-out ballot and participated during the actual community ballot exercise (see Community roll-out sequence ballot below).

#### Community roll-out sequence ballot

Especially within the project team, a lot of deliberation occurred regarding the order in which SMoTS would be rolled out. Scientific concerns, particularly about the randomness of the intervention process, were of overriding importance in this realm, but at the same time it was critical that the community would agree that the roll-out strategy was reasonable and fair.

Therefore, although drawing a sequence which would maximise the ability to measure an effect of the intervention was of utmost importance for the study, it was also necessary to develop a formula for selecting a sequence that was acceptable to community members. During discussions with various project stakeholders, among them project staff, members of the CBO and CAB, various approaches to balloting were introduced, discussed and simulated. Most of them were later dismissed because they were seen as unfair since they gave a perceived advantage to either some of those involved in the ballot process or some parts of the island. This was perceived to have the potential to reduce the credibility of the project and negatively impact acceptability with community members particularly those who would receive SMoTS later than the others.

Ultimately, based on insight from stakeholders, the project used a blind ballot approach where many possible roll-out sequences were computer-generated. Nine complete sequences (one starting in each metacluster) were presented to community members for selection according to a blind ballot. During the ballot, nine community members – one from each of the nine metaclusters – first picked a sealed number from numbers 1–9. The person who picked number one then picked a sealed envelope from nine unmarked envelopes each containing a different roll-out sequence. The sequence this person picked was the one the project followed. This approach was participatory for community residents and was perceived as a fair process. The ballot was conducted in a community forum. Community members who did not attend the balloting event were initially confused about the procedure but later on, following discussions with other community members and project staff, considered it fair and transparent.

#### Selection of houses to install the SMoTS

Once the project-initiated baseline demographic surveillance census of the island was completed, the number of houses was discovered to be much higher than earlier research had shown [29]. This meant that the project could only provide a SMoTS to each homestead rather than to each individual house. This led to a scenario where the project needed a system to determine the one house to install the SMoTS in cases where a homestead had more than one house.

Initially, the project anticipated using a balloting approach to select houses because this system would ensure a variety of houses were selected in different homesteads which would be representative of the mixture of houses on the island. However, it was important to choose a method that would show transparency of the selection process to residents. Therefore, based on insights from discussions with a section of project stakeholders and with the project CAB members, it was agreed that consensus among the members of the homestead would be the more socially acceptable method by the community. An approach for determining the house using a ballot would only be used where consensus among household members did not lead to the selection of one house to install. Table 2 shows a synthesis of the influences to the social and technical design of the intervention.

**Table 2:** **A synthesis of influences to the technical and social aspects of the intervention**

	**Technical influences**	**Social influences**
***Technical design features***		
1. Removal of carbon dioxide from the blend.	Need for daily replenishment of molasses mixture in all houses to ensure the same blend of odours in all houses.	Mobilisation of women to distribute molasses.
	Cost of procuring molasses.	
	Disposal of by-products of fermentation	
2. Change from fabric to metal trap cone.	The textile used absorbed the odour cues.	
3. Change to trap with rigid plastic base with fine mesh that allowed passage of odorant cues.	Need to increase airflow into the mosquito trap.	More appealing to end users.
4. Change of metal trap cones to plastic.		Researchers’ and residents’ concerns over theft of metallic SMoTS parts.
		Plastic cones cheaper than metal ones.
5. Inclusion of a port for phone charging.		Researchers wishes to provide a direct additional benefit to research participants.
***Social design features***		
1. Community roll-out sequence ballot	Need to maximise possibility of detecting effect of the intervention in complex island geography.	Scientists need for the roll-out to be legitimate and transparent in the eyes of the community.
		Community wishes to have an input in decision making.
2. Creation of CAB	Channel of communication for development of project and problem solving.	Scientists’ need to keep community involved and interested.
3. Choice of consensus method to select house to install with SMoTS in homesteads with multiple houses.		Community wishes to have a say and scientists wish to involve community members in decision making.
		Number of houses in a homestead.

## Discussion

Numerous studies have shown that successful innovations are usually based on an integration of technological and other ideas and insights from not only scientists, but also from users, intermediaries, and other societal agents. This shows the crucial role of empirical evidence in tailoring interventions to local settings. Typically, technological designs are negotiated achievements involving many parties [36]. The design process is the place where the various actors interested in a technology first share their ideas about the technology. Their diversity guarantees that the design represents many interests.

We looked at the intervention through the mutual shaping approach and this provided a more encompassing account of the impact of the joint processes of technical and social contexts. Our findings show how the various project stakeholders, including project staff, collaborators and community members, simultaneously pursued interdependent technological transformations and social interests. We see how in the on-going process, partial outcomes in the technological domain influenced social events at a later phase and vice versa.

Social shaping of technology is the way in which objects are changed because of their circumstances. Some technologies may require particular social relations to accompany them. In this project, considerations of a social nature also fed into the processes of deciding on the most practical odour bait for attracting malaria mosquitoes and during the re-design of the mosquito trap. Working with a blend without carbon dioxide provided much convenience in use and distribution for the researchers and residents. The cones of the mosquito traps were initially made of metal but were later changed to plastic since metal, although durable, would increase risk of theft. An additional advantage was that the use of a plastic cone made the trap more affordable without compromising its durability.

It has been argued that system innovation projects must enable the challenging and change of presumptions, current practices, and the underlying institutions, either in the design of a project or in its management [37]. In these reflexive undertakings institutions and their relations are not conceived as givens, but as objects of scrutiny and change. Initially during this intervention, community engagement was mainly channelled through a CBO operating in the community and that already carried out malaria-related work. This approach seemed appropriate but the project later on realised that the approach was not sufficient in representation of all community segments. Findings of other studies have shown that collaborations which are not representative of community-wide interests are a potential problem for participatory research [38,39]. The project consequently devised a CAB that was more deliberately representative in its nomination of members, guidelines and constitution. While there are strong philosophical reasons to involve diverse people and organisations in collaborative research efforts, broad engagement is also needed to strengthen the capacity of the community to identify, understand, and solve complex problems [40]. Partnerships with many different kinds of participants have a greater variety of nonfinancial resources to create synergy than those with few homogenous partners. This approach to creating and structuring sets of principles for community engagement is recommended as it recognises the specific local context and project [39].

Collaboration between researchers, the research community and the development of CAB have been identified as important issues in public health [41,42]. Involving the research community in decision making through a range of social research methods has been important in our research. We designed and simulated the community ballot with engagement of the CAB and input their ideas into the final approach to balloting. CABs are one strategy for establishing partnerships between researchers and host communities to promote community consultation in socially sensitive research [43,44]. In our study, the participation and input of community representatives has helped to solidify the partnership between the researchers and the research community.

Choosing a sequence for rolling out SMoTS involved a process of social and statistical cost and benefit analysis of sorts. We considered a method that would provide statistical power for measuring effects of the intervention and issues of social acceptability to accommodate the wishes of the residents. The method used also had to be practical as far as the geography of the island is concerned and was based on metaclusters earlier on defined by the project. Informed by insights from consultations with project stakeholders, we used a method that enabled participation of a community member from each of the metaclusters. In addition to involving community members in the ballot, we drew nine different possible sequences with each beginning installation of SMoTS at a different metacluster. This ensured each of the nine metaclusters had an equal chance of coming first and at the same time met the requirements of the scientific aspects of the intervention, namely to measure the impact on malaria in the community.

Social acceptability also played important roles in the method chosen to select the house in a homestead to install the SMoTS. While the project had initially considered randomisation at the homestead level because this would ensure all sorts of houses were included in the sample, it was important to use a formula whose outcome would not be contested by members of the homestead. We therefore selected a consensus approach among homestead members to select the house to install in homesteads with multiple houses.

## Conclusion

Our analysis has shown that the process of arriving at a more mature and better adapted technical and social design of the malaria control intervention involved a range of interactions, in which feedback from the technical and social environment were incorporated in the design and re-design and implementation strategy during the initial phases of the intervention. In generating this feedback, social science and natural science research were mutually useful and instrumental. To look at interventions this way requires a different role for research and a different perspective on innovation where innovation is more than the technical aspect. Feedback obtained from action research was used to not only see the workings of, but to also re-design the intervention. This approach therefore requires that research is designed in such a way that enables obtaining feedback from both aspects.

We argue that a mutual shaping perspective is well suited to capture the complexity and unpredictability of the interactions between technological features and social issues. Looking at intervention projects as niches that may evolve towards system innovation helps to reveal interrelations between the various technical and social aspects. The insights gained from this can be used to strengthen the designs of both the social and technical aspects of the intervention. This evidence-based re-design contributes towards aligning the innovation and therefore improves the survival chances of the innovation.

## Abbreviations

CBO: Community-Based Organisation
CAB: Community Advisory Board
KEMRI: Kenya Medical Research Institute
LED: Light-emitting diode
NGO: Non-governmental Organisation
SMoTS: Solar-powered mosquito trapping system
USB: Universal serial bus
